# Disseminated Histoplasmosis in Persons Living with HIV, France and Overseas Territories,1992–2021

**DOI:** 10.3201/eid3107.241931

**Published:** 2025-07

**Authors:** Mathieu Nacher, Esaïe Marshall, Firouze Bani-Sadr, Sandrine Peugny, Blandine Denis, Elise Ouedraogo, Sebastien Gallien, Agnes Meybeck, Ugo Françoise, Antoine Adenis, Nicolas Vignier, Pierre Couppié, Pierre Sellier, Sophie Grabar

**Affiliations:** Centre Hospitalier Universitaire de Guyane, Cayenne, French Guiana (M. Nacher, A. Adenis, N. Vignier); Université de Guyane, Cayenne (M. Nacher, A. Adenis, P. Couppié); Santé des Populations en Amazonie, Cayenne (M. Nacher, A. Adenis, P. Couppié); Institut Pierre Louis d’Epidémiologie et de Santé Publique, Paris, France (E. Marshall, S. Grabar); Centre Hospitalier Universitaire de Reims, Reims, France (F. Bani-Sadr); Centre Hospitalier Universitaire de Guadeloupe, Les Abymes, Guadeloupe (S. Peugny); Hôpital Saint Louis, Paris (B. Denis); Centre Hospitalier Universitaire La Pitié Salpêtrière, Paris (E. Ouedraogo); Hôpital Henri-Mondor, Créteil, France (S. Gallien); Centre Hospitalier de Tourcoing, Tourcoing, France (A. Meybeck); Hôpital Saint Antoine, Paris (U. Françoise); Hôpital Avicenne, Bobigny, France (U. Françoise, N. Vignier); Université Paris Cité, Paris (N. Vignier); Université Sorbonne Paris Nord, Paris (N. Vignier); Centre Hospitalier Universitaire de Guyane, Cayenne (P. Couppié); Hôpital Lariboisière, Paris (P. Sellier); Hôpital St Antoine, Paris (S. Grabar)

**Keywords:** histoplasmosis, HIV/AIDS and other retroviruses, *Histoplasma* spp., fungal infection, fungi, incidence, global burden, Americas, Caribbean, Asia, Africa, France

## Abstract

Disseminated histoplasmosis is a major issue among persons with advanced HIV in the Americas; it might also affect persons in sub-Saharan Africa, the Caribbean, and Asia and can be mistaken for other infections. By using 1992–2021 data from the French hospital database on HIV, we analyzed 198,798 persons with HIV follow-up in France and its overseas territories, identifying 553 (2.8/1,000 person-years) first episodes of disseminated histoplasmosis. Incidence rates varied by site of follow-up: 9.41 in French Guiana, 0.76 in Guadeloupe, 0.62 in Martinique, and 0.079 in mainland France. Incidence rates in France also varied between regions of origin or travel: 4.73 for Central or South America, 1.36 for the Caribbean, and 0.19 for sub-Saharan Africa or Asia. Differences persisted after adjusting for age, sex, CD4 count, and viral load at baseline. Overall, incidence and early death have declined, likely because of antiretroviral drug rollout in France.

Histoplasmosis is common in the Mississippi and Ohio Valleys in the United States, and the disseminated form was classified as an AIDS-defining disease in 1987 (*1*,*2*). Although much of the early work on disseminated histoplasmosis in persons with HIV (PWH) took place in the United States, Central and South America have also recognized the effects in PWH (*3*–*5*). Disseminated histoplasmosis is the leading AIDS-defining infection and cause of death in French Guiana and possibly in other South and Central America countries (*6*). In most endemic countries, the nonspecific manifestation of histoplasmosis, the low awareness of its epidemiologic importance among clinicians, and the lack of diagnostic tools leads to delays in timely diagnosis and treatment. The problem of histoplasmosis also seems to be ignored in Africa and Asia, where *Histoplasma capsulatum* is endemic (*7*–*9*). In the Caribbean, the extent of histoplasmosis is unclear (*10*). Given the movement of people between endemic zones and Europe, the number of cases in hospitals in France and its overseas territories might not be negligible.

The surge of disseminated histoplasmosis was closely linked to the HIV epidemic and the large numbers of PWH with advanced HIV disease. However, because antiretroviral drug therapy has been recommended for all PWH, the epidemiology is likely to have evolved with the gradual decrease in the proportion of severely immunocompromised patients (*11*). We have located few reports to date assessing this evolution.

For >30 years, the Agence Nationale de Recherche sur le SIDA et les Hépatites (ANRS) cohort 4 (CO4) French hospital database on HIV (FHDH), a large national hospital cohort, collects data from hospitals throughout France and, of note, from hospitals from overseas territories of France in South America (French Guiana), the Caribbean (Martinique and Guadeloupe), and the Indian Ocean (Reunion Island and Mayotte) (*12*). The cohort includes many PWH coming from a wide range of countries that are known as or suspected to be endemic for histoplasmosis (*7*,*8*,*13*,*14*). The different exposure to *Histoplasma* among PWH, combined with most being cared for by university hospitals with diagnostic tools capable of testing for fungal pathogens, enables assessment of the incidence of disseminated histoplasmosis according to the territory of residence or origin of PWH.

We used the ANRS CO4 FHDH to study the incidence of disseminated histoplasmosis in territories of France and the main factors associated with the disease and its prognosis in PWH during 1992–2021. We focused on the national or regional origins of PWH with disseminated histoplasmosis.

## Methods

### The ANRS CO4 FHDH 

Starting in 1989, the ANRS CO4 FHDH was an open hospital cohort of PWH. The cohort collects clinical, biological, and treatment data from adult PWH. The cohort is populated by data from 23 regional coordination committees for the fight against sexually transmitted infections and HIV (Comité de Coordination de la lutte contre le VIH et les IST [COREVIH]), including those from overseas territories (*12*). Each COREVIH has trained clinical research technicians collecting and verifying the quality of the local data and then transferring the data to the FHDH, where additional verifications are conducted to avoid inconsistencies and duplicates. Approximately 180 hospitals in France currently collect data for this cohort that includes 64% of PWH receiving care in France.

### Study Design

From data collected during January 1, 1992–December 31, 2021, we selected PWH with a confirmed HIV-1 or HIV-2 infection with >2 follow-up visits and 1 CD4+ T-cell measurement. Because diagnostic tools and first-line treatment for histoplasmosis are not always extensively collected in the FHDH, we contacted hospitals reporting cases of histoplasmosis over a 5-year period (2015–2020) to obtain additional information.

### ANRS CO4 FHDH Variables Studied

We extracted for the patients with (as main or secondary diagnoses, irrespective of the number of associated diagnoses) the B39 series of codes from the International Classification of Diseases, 10th Revision: B39, B390, B391, B392, B393, B394, B395, and B399, all defining histoplasmosis (*15*). We extracted date of birth, country of birth, and notion of residence in another country as a composite variable accounting for the geographic origin; we also extracted date of HIV diagnosis, date of first inclusion in the cohort, date of antiretroviral treatment initiation, first CD4 count, first viral load, date of histoplasmosis, date of follow-up, location of follow-up (mainland France, overseas territory), date of last news, and date of death. We classified AIDS by using the Centers for Disease Control and Prevention definition (*16*).

### Statistical Analysis

We computed incidence rates by using the Stata 16 (https://www.stata.com) suite of commands for survival analyses. We measured follow-up time from the date of inclusion in FHDH until a first diagnosis of histoplasmosis, death, last follow-up visit, or December 31, 2021. We computed crude incidence rates stratified for mainland France and different overseas territories and for different countries or regions of origin. We also computed the incidence rate of histoplasmosis by year in mainland France; in the French Antilles (Guadeloupe and Martinique); in French Guiana, Reunion Island and Mayotte; and by the following calendar periods: 1993–1996, 1997–2001, 2002–2006, 2007–2011, 2012–2016, and 2017–2021. For the initial viral load measurements, we categorized the variable as <20, 21–400, 401–1,000, 1,001–10,000, 10,001–100,000, >100,000 copies/mL, or missing. We introduced a missing category because viral load measurements only became available in 1997. For the first CD4+ T-cell count measurements, we categorized the variable as <50, 50–199, 200–349, 350–500, or >500 cells/mm^3^.

We identified factors associated with the risk for histoplasmosis by using a multivariable Cox proportional hazard model adjusted for first CD4 count at inclusion in the FHDH, categorized first viral load at inclusion in the FHDH, antiretroviral treatment status before histoplasmosis diagnosis, age at inclusion, sex, origin (categorized as France, Western Europe, Eastern Europe, North Africa, sub-Saharan Africa, Middle East, Asia, Australasia, Oceania, North America, South and Central America, and the Caribbean), and residence in mainland France or in 1 of the overseas territories. For participants treated in mainland France whose origin was 1 of the overseas territories, we classified their origin as French Guiana in South America; Martinique, Guadeloupe and Saint Martin in the Caribbean; or Reunion Island and Mayotte in the Indian Ocean. We verified the proportionality of hazards graphically. Age, a time-varying covariate, violated the assumption, and therefore we removed it from the Cox model and used it for stratification.

We computed overall mortality rates as the number of deaths divided by the total person-time since histoplasmosis diagnosis. We plotted Kaplan-Meier curves for the first 6 months after diagnosis of histoplasmosis to avoid misattributing death from another cause (*17*). For the subsample analysis, we conducted cross-tabulations between different hospitals and diagnostic methods used.

### Ethical and Regulatory Aspects

All persons enrolled in the cohort provided written informed consent for the use of their data for research purposes. The French data protection authority, the Commission Nationale de l’Informatique et des Libertés, first approved the cohort on November 27, 1991 (*18*). This authorization was later updated to comply with the new regulations, including the European Union’s general data protection regulation. On July 20, 2018, the ANRS CO4-FHDH cohort was also approved by the Comité d’Expertise pour les Recherches, les Études et les Évaluations dans le domaine de la Santé. On February 19, 2021, the cohort was also approved as a hospital data warehouse by the Commission Nationale Informatique et Libertés. On March 30, 2021, the ANRS CO4 FHDH cohort received the authorization to conduct research projects on the data warehouse by the Commission Nationale Informatique et Libertés (*18*). This study was approved by the scientific committee of the ANRS CO4 FHDH cohort.

## Results

### Description of Study Population

We analyzed 198,798 PWH (130,866 men, 66,625 women, and 1,307 other [persons whose personal identity is discordant from their birth sex]) during January 1, 1992–December 31, 2021, contributing 2,188,750 person-years of follow-up with a median follow-up of 9 (interquartile range [IQR] 2.6–7.2) years. The median age at inclusion in the FHDH was 34.4 years (IQR 28.8–41.9) years, and the median age at first episode of histoplasmosis was 41.3 (IQR 33.2–48.8) years. The median CD4 count in the cohort at last follow-up visit or on December 31, 2021, was 542 [IQR 361–753] cells/mm^3^, and 8.6% had CD4 counts <200 cells/mm^3^. Of live patients in the cohort, 22.5% were classified with AIDS at the last follow-up visit or on December 31, 2021.

Among the 198,798 PWH, the living area was recorded in 182,352 (91.7%) of cases. Most, 168,330 (91.9%) cases, were living in mainland France, 4,689 (2.5%) in French Guiana, 4,426 (2.4%) in Guadeloupe, 2,617 (1.4%) in Martinique, 1,863 (1%) in La Reunion Island, and 427 (0.2%) in Mayotte; information was missing for 16,446 (8.3%) cases. Patient origin was recorded in 183,650 (92.4%) cases, including 117,251 (58.9%) cases from mainland France, 49,103 (24.7%) from sub-Saharan Africa, 6,163 (3.1%) from Northern Africa, 5,765 (2.9%) from Central and South America, and 5,368 (2.7%) from the Caribbean; for 15,148 (7.6%) PWH, the information was missing.

Overall, there were 553 first incident episodes of disseminated histoplasmosis (Table 1). Apart from those first incident cases, 232 prevalent cases were reported at enrollment (171 men, 59 women, and 2 other), and in 92 cases (66 men, 25 women, 1 other), histoplasmosis occurred before enrollment in the FHDH. Those cases did not account for the incidence calculations.

**Table 1:** Summary characteristics of first disseminated histoplasmosis cases among persons living with HIV from the French Hospital Database on HIV, France and overseas territories, 1993–2021*

Variable	Value
Median age, y (IQR)	41.3 (33.2–48.8)
Sex	
M	352 (63.7)
F	197 (35.6)
Other†	4 (0.7)
Geographic origin	
Mainland France	166 (30)
Western Europe	1 (0.2)
Sub-Saharan Africa	83 (15)
Middle East	2 (0.3)
Asia	6 (1.1)
North America	1 (0.2)
South and Central America	189 (34.2)
Caribbean	105 (19)
Residence	
Mainland France	150 (28.8)
Martinique	17 (3.3)
Guadeloupe	34 (6.5)
French Guiana	318 (61)
Reunion Island	2 (0.4)
AIDS stage before histoplasmosis	
Yes	244 (44.1)
No	309 (55.9)
On antiretroviral treatment before histoplasmosis	
Yes	56 (10.1)
No	497 (89.9)
Median CD4, cells/mm**^3^** (IQR)	20 (6–53)
CD4/CD8 ratio >1 at time of histoplasmosis	
Yes	29 (5.7)
No	479 (94.3)
Median viral load at time of histoplasmosis, copies/mL (IQR)	16,480 (485–205,000)

*Values are no. (%) except as indicated. IQR, interquartile range †Other indicates that personal identity is discordant from birth sex.

For the 553 first episodes of disseminated histoplasmosis, median CD4 count was 171 (IQR 6–53) cells/mm^3^, and 89.9% of PWH were not on antiretroviral treatment when disseminated histoplasmosis occurred. Nearly two thirds (61%) of PWH were followed in French Guiana, and 15% of PWH originated from sub-Saharan Africa. No cases were reported from Eastern Europe, North Africa, Australasia, or Oceania.

### Diagnostic and Treatment Characteristics of 2015–2020 Samples

We identified 138 PWH to contact for additional information and received 90 responses with the requested information. Of those who responded, diagnosis was confirmed by PCR for 11 PWH (12.2%), direct examination for 29 PWH (32.2%), culture for 60 PWH (66.6%), and pathology for 35 PWH (38.8%). Antigen detection testing was not available.

Patients received induction treatment with liposomal amphotericin B in 61 (72.2%) cases (52.2% alone, 20% in combination with itraconazole) and with itraconazole alone in 25 (27.8%) cases. Information about treatment was missing in 4 cases. There was no statistical difference between hospitals (p = 0.36).

### Subgroups Associated with Incidence Variations

**Figure 1:**
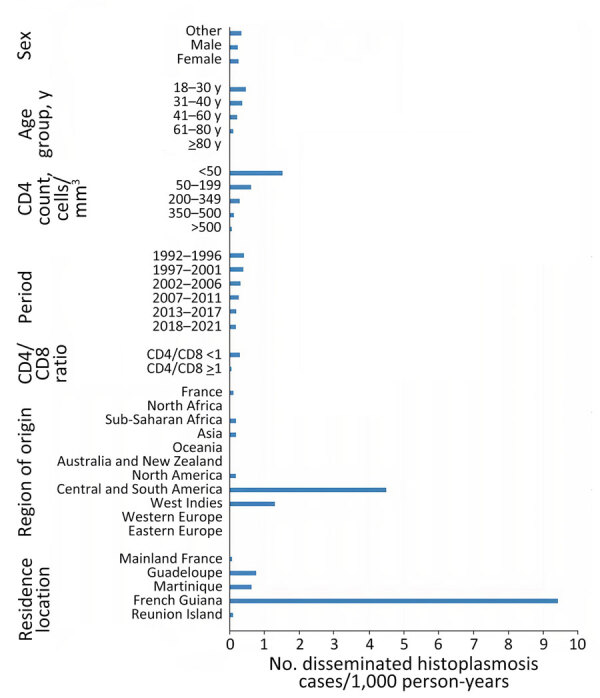
Incidence rate of disseminated histoplasmosis in persons living with HIV from France and overseas territories of France by different subgroups,1992–2021. Other sex refers to persons whose personal identity is discordant from their birth sex.

We organized specific incidence rates for disseminated histoplasmosis according to different subgroups (Figure 1). Younger patients, persons with low CD4 counts, persons originating from Central and South America and the Caribbean, and persons living in French Guiana or the French Antilles had higher incidences than other groups. The incidence rate was 9.41/1,000 person-years in French Guiana, 0.76/1,000 person-years in Guadeloupe, 0.62/1,000 person-years in Martinique, and 0.079/1,000 person-years in mainland France. The incidence rate was 4.73/1,000 person-years for PWH originating from Central and South America, 1.36/1,000 person-years for PWH originating from the Caribbean, and 0.19/1,000 person-years for PWH originating from sub-Saharan Africa or Asia.

After adjustments, a lower initial CD4 count, a higher initial HIV viral load, and residing in French Guiana or in the French Antilles (Guadeloupe/Martinique) were associated with higher hazard ratios of histoplasmosis. Regarding country or region of origin, PWH from Central and South America, Asia, and sub-Saharan Africa had an independently greater hazard ratio of histoplasmosis than those from mainland France (Table 2).

**Table 2:** Crude and adjusted hazard ratios for disseminated histoplasmosis in persons living with HIV from France and overseas territories of France,1992–2021*

Variable	Person-time, y	First events	Hazard ratio (95% CI)		p value
			Crude	Adjusted†	
Sex					<0.0001
M	1,438,462	352	Referent	Referent	
F	741,990	197	1.1 (0.92–1.30)	0.50 (0.40–0.63)	
Initial CD4 count, cells/mm^3^					
<50	51,925	79	15.40 (11.10–21.38)	8.94 (6.26–12.77)	<0.0001
51–199	181,448	113	8.11 (6.00–10.96)	4.63 (3.38–6.35)	<0.0001
200–349	326,745	97	4.09 (3.00–5.58)	2.60 (1.88–3.58)	<0.0001
350–500	406,760	52	1.78 (1.24–2.56)	1.36 (0.94–1.97)	0.10
>500	948,770	68	Referent	Referent	
Initial viral load, copies/mm^3^					
<20	1,069,688	94	Referent	Referent	0.24
21–400	240,498	51	2.40 (1.70–3.37)	1.27 (0.84–1.93)	0.001
401–1,000	53,468	22	4.68 (2.94–7-44)	2.42 (1.40–4.17)	0.003
1,001–10,000	135,410	35	2.93 (1.99–4.33)	1.89 (1.23–2.91)	0.005
10,001–100,000	143,257	51	4.04 (2.87–5.69)	1.78 (1.18–2.67)	<0.0001
>100,000	64,702	79	13.79 (10.22–18.61)	4.20 (2.88–6.13)	<0.0001
Missing	485,100	221	5.21 (4.08–6.66)	3.26 (2.43–4.36)	
Residence					
Mainland France	1,889,675	150	Referent	Referent	<0.0001
Guadeloupe	44,325	34	9.35 (6.44–13.57)	12.15 (7.42–19.91)	<0.0001
Martinique	27,112	17	7.69 (4.65–12.69)	11.95 (6.47–22.07)	<0.0001
French Guiana	33,767	318	102.88 (84.64–125.06)	111.43 (79.18–156.82)	0.36
Reunion Island	19,482	2	1.28 (0.31–5.16)	1.90 (0.46–7.73)	
Origin					
France	1,437,390	166	Referent	Referent	0.47
Western Europe	58,573	1	0.16 (0.02–1.15)	0.48 (0.06–3.50)	NA
North America	5,414	1	1.67 (0.23–11.94)	NA	<0.0001
Central and South America	39,905	189	31.88 (25–70–39.54)	2.57 (1.84–3.58)	0.36
Caribbean	76,849	105	13.30 (10.38–17.03)	1.17 (0.82–1.65)	<0.0001
Sub-Saharan Africa	435,761	83	2.00 (1.52–2.62)	4.52 (3.18–6.42)	NA
Middle East	9,368	2	2.14 (0.53–8.66)	NA	<0.0001
Asia	30,572	6	1.99 (0.88–4.51)	5.50 (2.21–13.69)	NA

*NA, not available. †Model stratified on age group (age categories are time-varying and violate hazard proportionality assumption); excluded by Stata (https://www.stata.com).

### Incidence and Trends According to Places of Residence

**Figure 2:**
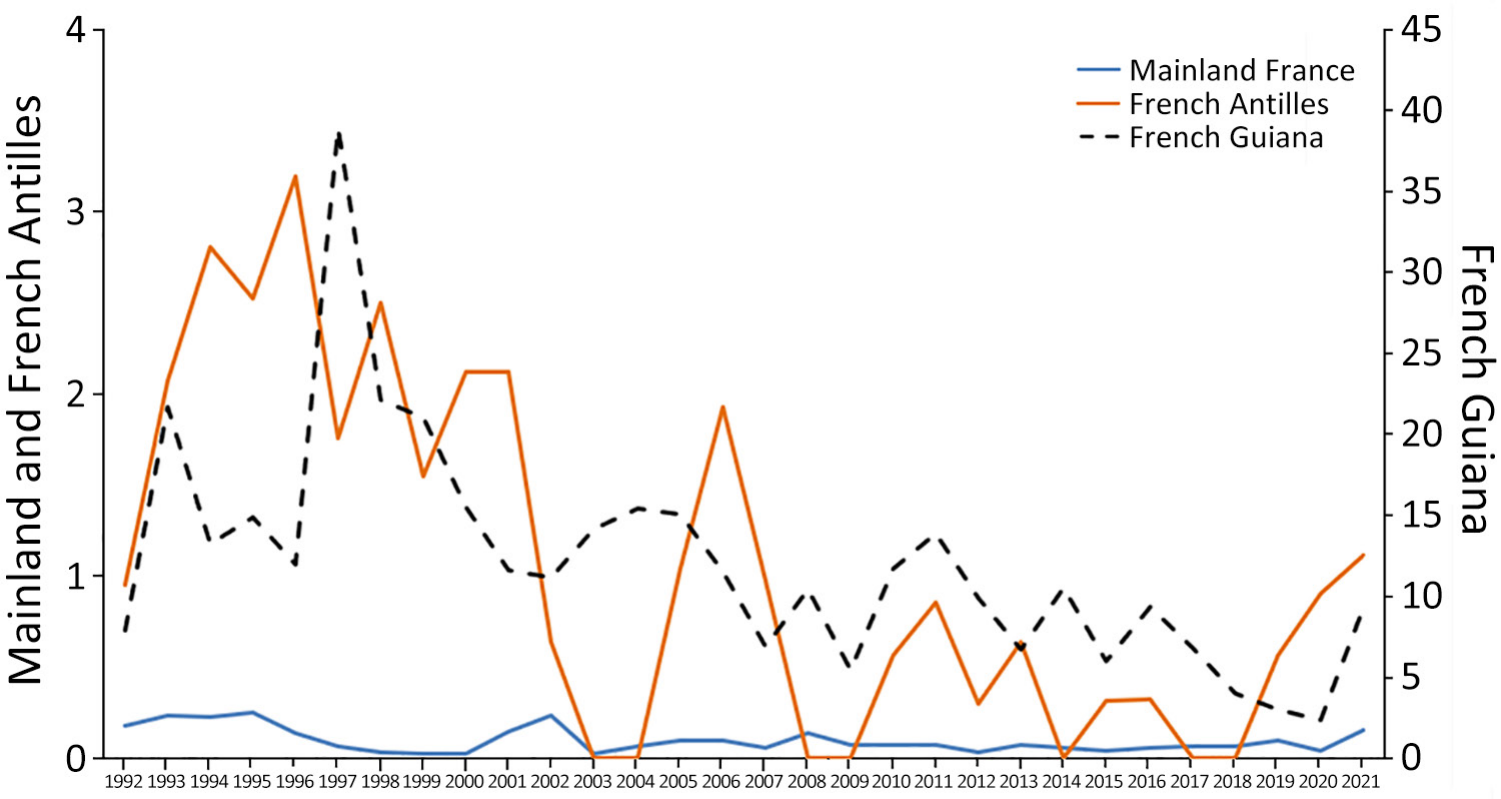
Evolution of the annual incidence rate of disseminated histoplasmosis per 1,000 person-years in mainland France, the French Antilles, and French Guiana, 1992–2021. Scales for the y-axes differ substantially to underscore patterns but do not permit direct comparisons.

The crude overall incidence of disseminated histoplasmosis was 0.25/1,000 person-years, and we observed an incidence decline of histoplasmosis in mainland France, the French Antilles, and in French Guiana (Figure 2). No cases were reported in the Antilles in 2003, 2008, 2014, or 2017. Incidence rates were considerably higher in French Guiana and in the French Antilles than in mainland France. Incidence declined over time in all 3 locations. In 2020, there seemed to be a reversal of the trend in French Guiana, the French Antilles, and mainland France.

### Mortality Rates in PWH with Disseminated Histoplasmosis

**Figure 3:**
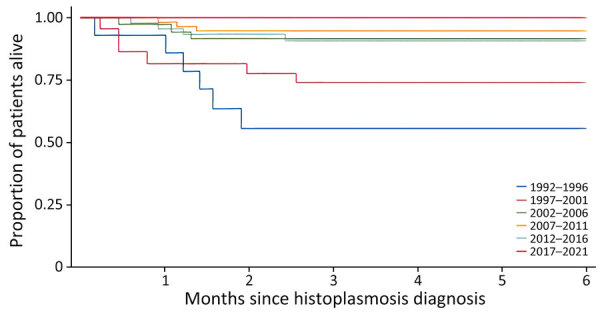
Kaplan-Meier survival curve representing deaths among disseminated histoplasmosis in persons living with HIV, by calendar time periods, France and overseas territories, 1992–2021.

Of the 553 PWH with histoplasmosis, 128 died. The incidence rate of death was 2.98 (95% CI 2.20–4.04)/100 person-years. Most deaths occurred within 6 months of the diagnosis of histoplasmosis; 17.7% deaths overall occurred by 1 month after diagnosis. Over time, there was a marked reduction of early death after histoplasmosis, from 51% during 1992–1996 to 3% during 2017–2021 (p<0.0001 by log-rank test) (Figure 3).

## Discussion

We found the incidence of disseminated histoplasmosis in PWH was much higher (>100-fold) among persons residing in French Guiana and among persons residing in the French Antilles (≈10-fold) than among persons residing in mainland France. Analysis of incidence by country or region of origin revealed persons originating from Central and South America and from the Caribbean were at greatest risk for disseminated histoplasmosis. Of note, the incidence of disseminated histoplasmosis was higher among PWH who had lived in sub-Saharan Africa or Asia than those originating from mainland France who had not lived elsewhere.

We demonstrated that, over the 30-year study period, with the increased proportion of virologically controlled HIV and with high CD4 count PWH, the incidence of disseminated histoplasmosis has decreased everywhere by 2–3-fold. However, the decrease of the incidence was the most critical in French Guiana and the French Antilles. Early death after a first episode of histoplasmosis was very high in the 1990s but also substantially decreased over time, as shown previously (*19*).

This study provides an overview of the risk for disseminated histoplasmosis in PWH having lived with or currently living with different levels of exposure. Because of the lack of diagnostic tools, histoplasmosis has often been overlooked in Latin America, the Caribbean, and in sub-Saharan Africa, where the reservoir of PWH with advanced HIV is the largest (*7*–*9*,*20*). In this cohort, around one quarter of PWH originated from sub-Saharan Africa. We hypothesized if the incidence of histoplasmosis is so much higher in PWH originating from Latin America and the Caribbean than from other regions, it is because histoplasmosis is endemic in the former. New studies in different parts of the world seem more frequent since the Manaus declaration and the greater availability of antigen detection tests (*5*,*21*–*32*). However, a systematic review found only 6 studies using antigen detection to determine prevalence in PWH in Latin America and 9 in sub-Saharan Africa, but even fewer from Asia, North America, Europe, and Australia (*33*). Studies using biobanked samples could enable assessment of the extent of histoplasma in countries without any data. The study also shows the dramatic decrease of incidence and early death from disseminated histoplasmosis with virological control and high CD4 count in PWH.

The first limitation of our study is that our cohort could reflect the global epidemiology of histoplasmosis, but we cannot rule out biases because of varying durations and levels of exposure. Second, the case records, the types of diagnostic tools (sometimes by using the European Organization for Research and Treatment of Cancer’s Mycoses Study Group criteria [*34*]), and treatment schedules were not standardized and may vary from 1 clinician to another or from 1 hospital to another. Third, in our 2015–2020 sample, none of the diagnoses were on the basis of antigen detection tests (unavailable in France), despite most PWH in our cohort being followed in university hospitals. Fourth, in our 2015–2020 sample, the first-line treatment was liposomal amphotericin B in about three quarters of cases, which, according to World Health Organization guidelines (*35*), implied most cases were severe or moderately severe. We did not observe such severity in French Guiana, which has the largest histoplasmosis cohort (*17*). Fifth, the cause of death was not always precisely defined so similar to previously published reports, so we could only assume that early deaths were because of histoplasmosis (*17*). Finally, differing from the residence, which enables categorizing a patient in a potential endemic zone, the country or region of origin is a more inconclusive variable: we assumed that the patient born or having spent time in a potential endemic zone (or not) influenced the probability of acquiring histoplasmosis. In 2019, a doctoral thesis based on the French National Mycology Reference Laboratory data identified 107 cases of histoplasmosis (61% in PWH); all either came from or had traveled to endemic areas, mostly the Americas, western sub-Saharan Africa, and Asia (*36*). Despite those potential limitations, the large size and longitudinal design provide precious information about a major global pathogen.

This article underscores that the Americas have the highest prevalence of histoplasmosis (*37*) and suggests there are cases elsewhere, perhaps fewer than the number of cases found in simulations (*9*). Because of the burden of HIV in sub-Saharan Africa or Asia, even modest prevalence could lead to substantial illness and death in those regions. Finally, with the antiretroviral-for-all recommendations for PWH (*11*), the effects of histoplasmosis could decline worldwide. Because the prevalence of histoplasmosis appears related to advanced HIV disease, HIV testing and proactive care are potent tools to decrease the prevalence of histoplasmosis.

In conclusion, this article reveals the incidence of disseminated histoplasmosis was highest in PWH whose follow-up was in French Guiana and the French Antilles. Among those living in mainland France, incidences were highest in PWH coming from or having stayed in Central and South America, followed by Caribbean. PWH originating from sub-Saharan Africa or Asia had higher incidences than those originating from mainland France. Because having spent time in each region can correspond to a range of durations, studies should directly estimate the incidence of histoplasmosis among PWH in each region. During the 30-year study period, the incidence and early death rates of histoplasmosis have steadily decreased, likely because of broadening use of antiretroviral drugs.
